# Comparing the Clinical Manifestations of Bell’s Palsy between Pre-COVID-19 Pandemic and COVID-19 Pandemic Periods

**DOI:** 10.3390/jcm12041700

**Published:** 2023-02-20

**Authors:** Gang Won Choi, Dong Keon Yon, Yong Sung Choi, Jinseok Lee, Ki Ho Park, Young Ju Lee, Dong Choon Park, Sang Hoon Kim, Jae Young Byun, Seung Geun Yeo

**Affiliations:** 1 Department of Otorhinolaryngology Head & Neck Surgery, Kyung Hee University School of Medicine, Kyung Hee University Medical Center, Seoul 02447, Republic of Korea; 2 Center for Digital Health, Medical Science Research Institute, Kyung Hee University School of Medicine, Kyung Hee University Medical Center, Seoul 02447, Republic of Korea; 3 Department of Pediatrics, Kyung Hee University School of Medicine, Kyung Hee University Medical Center, Seoul 02447, Republic of Korea; 4 Department of Biomedical Engineering, Kyung Hee University, Youngin 17104, Republic of Korea; 5 Division of Infectious Diseases, Department of Internal Medicine, Kyung Hee University School of Medicine, Kyung Hee University Medical Center, Seoul 02447, Republic of Korea; 6 Department of Obstetrics and Gynecology, Kyung Hee University School of Medicine, Kyung Hee University Medical Center, Seoul 02447, Republic of Korea; 7 Department of Obstetrics and Gynecology, St. Vincent’s Hospital, The Catholic University of Korea, Suwon 14647, Republic of Korea

**Keywords:** COVID-19, Bell’s palsy, comparison

## Abstract

Background: COVID-19 has been shown to affect the onset and severity of various diseases. We examined whether the clinical characteristics of Bell’s palsy differed between before and during the COVID-19 pandemic. Methods: From January 2005 to December 2021, 1839 patients were diagnosed and treated for Bell’s palsy at Kyung Hee University Hospital. These patients were divided into a pre-COVID period group and COVID-19 period group, and the clinical characteristics of the two groups were compared. Results: There were 1719 patients in the pre-COVID period group and 120 patients in the COVID-19 period group. There were no between-group differences in sex (*p* = 0.103) or in the presence of underlying hypertension (*p* = 0.632) or diabetes (*p* = 0.807). Regarding symptoms, there were no significant between-group differences in otalgia, dizziness, tinnitus, hyperacusis, or hearing loss (*p* = 0.304, *p* = 0.59, *p* = 0.351, *p* = 0.605, and *p* = 0.949). There were also no significant between-group differences in electroneurography results (*p* = 0.398), electromyography results (*p* = 0.331), House–Brackmann Grade at visit (*p* = 0.634), or recovery rate after treatment (*p* = 0.525). Conclusions: Contrary to our expectation that Bell’s palsy cases during the COVID-19 pandemic would show different clinical features than those occurring before COVID-19, the present study found no differences in clinical features or prognosis.

## 1. Introduction

Facial nerve palsy directly affects quality of life, and patients with facial nerve palsy show higher rates of depression and social activity limitations than patients with other diseases [1]. Bell’s palsy, the most common type of facial palsy, may be caused by viral infections, ischemic vascular disease, blood circulation disorders, and autoimmune diseases. The clinical symptoms and changes in viral antibody titers observed in Bell’s palsy patients suggest that the condition is caused by a virus; however, to date, no such virus has been isolated from patient serum [2]. Viruses that can cause facial paralysis include herpes simplex virus, influenza virus, adenovirus, cytomegalovirus, echovirus, enterovirus, Epstein–Barr virus, cytomegalovirus, human herpesvirus, human immunodeficiency virus, mumps, rubella, poliomyelitis, and varicella zoster virus [3,4]. Relatively few systematic studies have examined whether severe acute respiratory syndrome coronavirus 2 (SARS-CoV-2) can cause facial paralysis, but many suspected cases have been recently reported, and research on the relationship between clinical features is being conducted.

Coronavirus infectious disease-19 (COVID-19) is a respiratory syndrome caused by SARS-CoV-2 infection. Although coronaviruses typically cause only mild respiratory infections, such as the common cold in humans, the World Health Organization declared COVID-19 a global pandemic in December 2019, just 3 months after the first case was reported. The fatality rate for COVID-19 varies greatly by country and by patient age; the global fatality rate reported by the World Health Organization is 2.1%, with severe infection and death most often seen in elderly patients, immunocompromised patients, and patients with underlying diseases. As of 16 December 2022, more than 653,984,942 people worldwide had been confirmed to be infected with severe acute respiratory syndrome coronavirus 2 (SARS-CoV-2), and 6,667,620 people had died with the disease, for a fatality rate of 1.02%. In Korea, 27,995,726 people were confirmed to be positive for infection, and 31,232 died from COVID-19, for a fatality rate of 0.11% [5,6,7]. In addition to the direct effects of COVID-19 infection or vaccination, COVID-19 epidemic and quarantine rules have impacted the onset and prognosis of various other diseases. Here, we hypothesized that Bell’s palsy cases occurring before and during the COVID-19 pandemic would show different clinical manifestations and prognosis. We therefore examined whether the clinical manifestations and prognosis of facial palsy differed between before and during the COVID-19 epidemic.

## 2. Materials and Methods

### 2.1. Subjects

We retrospectively investigated 1839 patients who were diagnosed with facial nerve palsy and hospitalized between January 2005 and December 2021 at Kyung Hee University Hospital. Patients with intakes ranging from January 2005 to December 2019 were placed in the pre-COVID group, while those with intakes ranging from January 2020 to December 2021 were placed in the COVID-19 group. The age, gender, underlying diseases, accompanying symptoms, recovery rate, and clinical manifestations were compared between the two groups.

The inclusion criterion was a documented history of idiopathic facial paralysis. The exclusion criteria were as follows: congenital malformation, trauma, acquired immune disease, sequelae or complications of systemic disease, central nervous system disorders, polyneuropathy such as Guillain–Barre syndrome, recurrent Bell’s palsy, iatrogenic facial nerve palsy, neoplasms, Ramsay Hunt syndrome, otitis media, otologic surgery, bilateral facial palsy, metastasis, concomitant meningitis, myelitis, vasculopathy, and insufficient documentation or test results.

All patients received oral steroids and antiviral drugs immediately after diagnosis, and additionally received physical therapy or self-facial paralysis rehabilitation treatment. Oral corticosteroids were administered at 1 mg/kg based on the patient’s body weight for the first 4 days and then on a decreasing trend for 8 days thereafter. The degree of facial paralysis was evaluated based on the House–Brackmann (HB) classification method. Electroneurography (ENoG) and electromyography (EMG) were performed as neurological tests for facial palsy. ENoG was performed on the 4th or 5th day of facial paralysis, and needle EMG was performed on the 14th day of facial paralysis. Results were reported as the percentage of the maximal amplitude on the affected side divided by the maximal amplitude on the normal side. A poor ENoG result was defined as a >90% loss in amplitude, whereas a good result was defined as a ≤90% loss. The presence or absence of a blink reflex and needle EMG results were analyzed simultaneously, with outcomes classified as poor or good by the physical medicine and rehabilitation physicians. The absence of pathological spontaneous fibrillation activity was defined as a good outcome, whereas the presence of abnormal spontaneous activity or the absence of volitional activity was classified as a poor outcome [8]. Final HB grade I or II was defined as satisfactory recovery or favorable recovery, while a grade of III or higher was defined as incomplete or unfavorable recovery. The study protocol was approved by the Institutional Review Board of Kyung Hee University Hospital. Informed consent was not required, as this was a retrospective study (IRB No 2019-07-065).

### 2.2. Statistical Analysis

Continuous variables, such as age, were compared using t-tests. Categorical variables, such as initial and final HB grade, sex, diabetes mellitus (DM), hypertension (HTN), comorbid symptoms, ENoG, and EMG were compared using chi-squared or Fisher’s exact tests. Categorical variables were reported as a number (percentage), whereas continuous variables were reported as mean ± standard deviation. All statistical analyses were performed using IBM SPSS version 20.0 (IBM Corp., Armonk, NY, USA). Statistical significance was accepted at *p* < 0.05.

## 3. Results

Characteristics of patients in the pre-COVID period and COVID-19 period groups (Table 1).

Among the total of 1839 patients diagnosed with Bell’s palsy at Kyung Hee University Hospital from January 2005 to December 2021, 1719 were classified to the pre-COVID group and 120 to the COVID-19 group. There were slightly more females overall [847 males (46.0%) and 992 females (53.9%)] and in the pre-COVID period group (797 males and 922 females), but this parameter did not differ significantly between the pre-COVID and COVID-19 groups (*p* = 0.103). The age ranged from 0 to 88 years across all enrolled patients; the average age of all patients was 47.85 ± 16.67 years, that of the pre-COVID period group was 47.60 ± 16.70 years (46.80 ± 16.70 years for males and 48.20 ± 16.70 years for females), and that of the COVID-19 group was 51.45 ± 16.26 years (50.30 ± 16.32 years for males and 52.27 ± 16.21 years for females). There was no significant difference in age between the pre-COVID and COVID period groups (*p* = 0.062). Regarding the presence or absence of underlying hypertension and/or diabetes in the pre-COVID and COVID period groups, hypertension was found in 352 patients (20.4%) and 22 patients (18.3%), while diabetes was found in 216 patients (12.5%) and 16 patients (13.3%), respectively. These rates did not differ significantly between the two groups (*p* = 0.632 and *p* = 0.807, respectively). Regarding accompanying symptoms, the rates of otalgia, dizziness or vertigo, tinnitus, hyperacusis, and hearing loss did not differ significantly between the two groups (*p* = 0.304, *p* = 0.59, *p* = 0.351, *p* = 0.605, and *p* = 0.949, respectively).

### 3.1. Results of Electroneurography (ENoG) and Electromyography (EMG) (Table 2)

In the pre-COVID period group, 93.9% of ENoG results were good and 6% were poor. In the COVID period group, 95.8% were good and 4.1% were poor. There was no significant difference in ENoG results between the groups (*p* = 0.398). In the pre-COVID period group, 63.9% of EMG values were good and 36.0% were poor. In the COVID period group, 68.3% were good and 31.6% were poor. There was no significant difference in EMG results between the groups (*p* = 0.331).

**Table 2:** Comparison of ENoG and EMG results between pre-COVID-19 and COVID-19 periods.

Parameter	Pre-COVID-19 Period (*n* = 1719)	COVID-19 Period (*n* = 120)	*p*–Value
	1615 (93.9)	115 (95.8)	0.398
ENoG (Poor)	104 (6.0)	5 (4.1)	
EMG (Good)	1099 (63.9)	82 (68.3)	0.331
EMG (Poor)	620 (36.0)	38 (31.6)	

Electroneurography (ENoG); electromyography (EMG).

### 3.2. House-Brackmann Grade at First Visit (Figure 1)

In the pre-COVID period group, 57.3% of patients had HB grade II and III, 32.2% had HB grade IV, and 10.4% had HB grade V and VI. In the COVID period group, 61.7% of patients had HB grade II and III, 28.3% had HB grade IV, and 10.0% had HB grade V and VI. There was no significant difference between the groups (*p* = 0.634).

**Figure 1:**
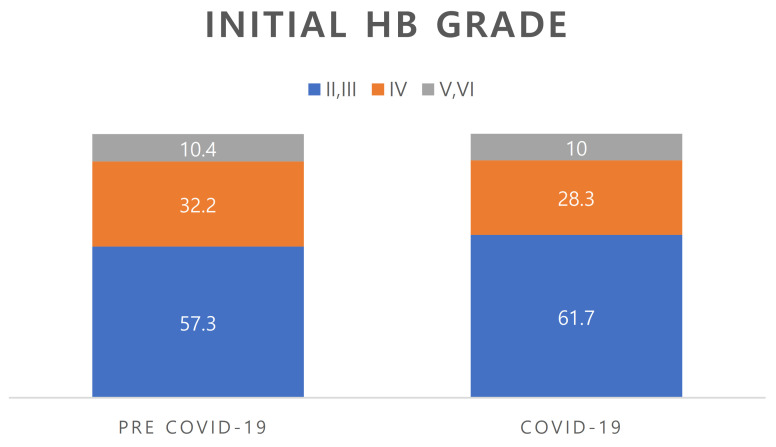
Comparison of the initial HB grade between pre-COVID-19 and COVID-19 periods.

No significant between-group difference was seen (*p* = 0.634). HB; House–Brackmann.

### 3.3. Recovery Rate (Figure 2)

In the pre-COVID period group, the final recovery rate to HB grade II or higher was 90.2%, and the rate of poor outcomes was 9.8%. In the COVID period group, the final recovery rate to HB grade II or higher was 91.7%, and the rate of poor outcomes was 8.4%. There was no significant difference between the groups (*p* = 0.525).

**Figure 2:**
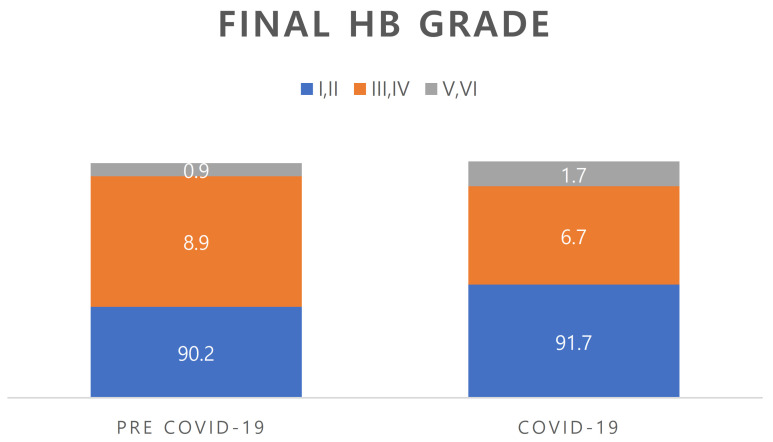
Comparison of the final HB grade between pre-COVID-19 and COVID-19 periods. No significant between-group difference was seen (*p* = 0.525). HB; House–Brackmann.

## 4. Discussion

Common symptoms of COVID-19 include fever, fatigue, nasal congestion, myalgia, loss of smell, dry cough, halitosis, hemoptysis, and respiratory difficulty. More severe symptoms include severe pneumonia, acute respiratory distress syndrome, sepsis, septic shock, and multiple organ failure, resulting in death [6]. Less common complications include hemophagocytic lymphohistiocytosis (HLH), vasculitis, and central retinal vein occlusion [9,10,11]. The pathophysiology associated with facial palsy is still unknown.

Available data are contradictory in regard to whether a link exists between SARS-CoV-2 infection and facial paralysis. Some reports have suggested that SARS-CoV-2 infection can cause facial paralysis. One study found that Bell’s palsy was the only major neurological manifestation observed in COVID-19 patients [12]. Another found that SARS-CoV-2 IgM and IgG were increased in patients presenting with Bell’s palsy during the COVID-19 pandemic [13]. A third study found that the incidence of facial paralysis was significantly higher during than before the COVID-19 pandemic, with a facial paralysis incidence rate of 1.73 [14]. However, other studies have generated contradictory findings: one failed to find a difference in the incidence of Bell’s palsy between before and during the COVID-19 pandemic, while another found no difference in the incidence of facial paralysis in children between before and during the COVID-19 pandemic [15,16]. Therefore, it remains unclear whether the incidence of Bell’s palsy increased during the COVID-19 pandemic, and the relationship between COVID-19 and Bell’s palsy is not known. Moreover, although there have been reports on the incidence of coronavirus infection and facial paralysis, nothing is known about whether the clinical symptoms, prognosis, and accompanying symptoms of Bell’s palsy patients differed between before and during the COVID-19 pandemic. Therefore, in the present study, we analyzed the clinical symptoms, prognosis, and accompanying symptoms of Bell’s palsy during the COVID-19 pandemic period and compared them with the period preceding the pandemic.

The present study found no significant difference in the sex distribution of patients with Bell’s palsy between before and during the COVID-19 pandemic. Two previous systematic reviews of recently reported cases found a slightly higher tendency for facial paralysis among males, but this difference was not significant [17,18]. However, in both the pre- and pandemic periods, there was no significant difference in the incidence of Bell’s palsy between males and females. In this study, facial paralysis presented roughly equally on each side of the face, with 29 patients presenting paralysis on the left side and 28 patients on the right side. Patients with unilateral facial palsy showed left and right involvement at approximately equal rates. Regarding the severity of paralysis, our pre-COVID period group comprised 57.3% HB grade II and III, 32.2% HB grade IV, and 10.4% HB grade V and VI, and the COVID period group comprised 61.7% HB grade II and III, 28.3% HB grade IV, and 10.0% HB grade V and VI. The severity of initial facial paralysis did not differ between the two groups (*p* = 0.634). The ENoG and EMG results also did not differ between the groups, indicating that patient prognosis did not differ between before and during the pandemic.

The above results collectively showed that, contrary to our expectations, there was no difference in the clinical aspects of Bell’s palsy patients between before and during the COVID-19 pandemic. The final recovery rate of the pre-COVID period group was 90.2% to HB grade II or higher (favorable outcome) and 9.8% for poor outcome, while the rates in the COVID period group were 91.7% and 8.4%, respectively. There was no significant between-group difference in this parameter (*p* = 0.525). These findings differ somewhat from those in the literature. In a systematic review of facial paralysis in patients with COVID-19 from 1 December 2019 to 21 September 2021, in 49 studies involving 74 facial paralysis patients, complete paralysis was observed in one case, and most cases had moderate-to-severe facial dysfunction. Positive treatment outcomes were seen in 83.5% of patients, whereas 14.9% showed nonsignificant recovery [17]. However, studies on SARS-CoV-2-associated cranial nerve mononeuropathies or polyneuropathies yielded different results. Fifty-six patients from 36 articles were included, and the following was seen: cranial nerve I involvement in three patients, cranial nerve II involvement in seven patients, cranial nerve III involvement in 15 patients, cranial nerve IV involvement in one patient, cranial nerve V involvement in six patients, cranial nerve VI involvement in 17 patients, cranial nerve VIII involvement in two patients, cranial nerve IX and X involvement in five patients each, cranial nerve XI involvement in no patient, and cranial nerve XII involvement in four patients. Facial nerve involvement was the highest in 29 patients, and SARS-CoV-2 was more involved than other cranial nerves. Twenty-four patients had Guillain–Barre syndrome (GBS) with cranial nerve involvement. The treatment prognosis was much lower than for general facial paralysis, with “complete recovery” seen in 21 patients and “partial recovery” seen in 30 patients, resulting in poor results for the COVID-19 [18]. Another study found that Bell’s palsy was the initial manifestation in 37% of cases, and in 63% of cases it developed after COVID-19 symptoms [19].

We can suggest several explanations for the differences between our results and those of the above-described study. First, if other cranial nerves in addition to the facial nerve are invaded, the treatment effect may differ from that seen in Bell’s palsy. Second, the previously reported studies included patients with facial palsy from other causes, such as GBS and Ramsay Hunt syndrome. Third, previous studies on facial paralysis caused by COVID-19 included patients with severe diseases that led to death; because such cases were not included in the present study, there may have been clinical differences. Fourth, the previous study was not single-institutional; rather, it was a systematic review that summarized cases from multiple institutions, and the data were therefore analyzed based on different standards.

Mechanistically, although we do not yet understand the pathophysiology underlying the link between SARS-CoV-2 and facial paralysis, several hypotheses have been proposed. First, the invasion of cranial nerves by SARS-CoV-2 is thought to occur via retrograde transport of viral particles to the brain after the virus is absorbed into the intracellular space of neurons in a distal location. This hypothesis is supported by an autopsy study of 43 patients who died of COVID-19, in whom viral proteins were detected in cranial nerves and isolated brainstem cells derived from the lower brainstem [20]. Moreover, in experimental studies, SARS-CoV-2 was found to be transported retrogradely to the CNS within the axons of cranial nerves [21]. The second hypothesis is that the immunological response to the virus secondarily affects neuronal structures due to epitope mimicry, as seen in GBS [22]. The third hypothesis is that drugs given to treat COVID-19 may have neurotoxic side effects that damage the cranial nerve. Drugs that are known to cause neuropathy and have been frequently administered to COVID-19 patients include daptomycin [23], linezolid [24], lopinavir [25], ritonavir [26], hydroxychloroquine [27], cisatracurium [28], clindamycin [29], tocilizumab [30], and glucocorticoids [31]. Fourth, neural tissues such as neurons, oligodendrocytes, and astrocytes widely express ACE2, meaning that nerve tissues are vulnerable to direct invasion of SARS-CoV-2. After invading nervous tissue, SARS-CoV-2 alters the cellular transport function and promotes transport from one neuron to another [32,33]. Fifth, the glycoproteins on the surfaces of coronaviruses are similar to the glycoconjugates of human tissue, and thus will tend to engage in “molecular mimicry”. Therefore, antibodies formed against viral surface glycoproteins act against glycoconjugates in nerve tissue and cause nerve damage [34,35].

The present study has several limitations. First, because it was a retrospective study, there was a large difference in the number of cases between the two groups. This could have influenced the results of the statistical analysis. Second, we do not know whether the Bell’s palsy that occurred during the COVID-19 pandemic was due to COVID-19 infection or other causes. Third, no epidemiologic investigation to date has examined whether social distancing, hand washing, mask wearing, and prohibition of group activities has a direct effect on the occurrence of Bell’s palsy in COVID-19 pandemic period. Fourth, the literature suggests that there may be between-country differences in the clinical aspects of facial palsy. This study examined Bell’s palsy at one institution in Korea, and the clinical findings from our population could differ from those in other countries. Therefore, additional research data on cross-country and regional differences in the incidence and prognosis of COVID-19 and Bell’s palsy are needed. Fifth, all of the patients in the COVID-19 period group in the present study received corona tests on samples from the nose and throat to test for the presence of COVID-19 infection before hospitalization, and all were negative. However, polymerase chain reaction (PCR), reverse transcriptase (RT)-PCR, and serology were not performed.

## 5. Conclusions

The present study found no differences in the clinical features, prognosis, and treatment effects of Bell’s palsy before and during the COVID-19 pandemic. However, in the future, a global epidemiological survey or systematic review and meta-analysis should be conducted to address whether the pandemic affected the occurrence and prognosis of Bell’s palsy.

## Figures and Tables

**Table 1:** Demographics.

Parameter	Pre-COVID-19 Period (*n* = 1719)	COVID-19 Period (*n* = 120)	*p*-Value
Age, yr	47.60 ± 16.70	51.45 ± 16.26	0.062
Sex, *n* (%)			
Male	797 (46.3)	50 (41.7)	0.103
Female	922 (53.6)	70 (58.3)	
Underlying disease			
Hypertension, *n* (%)	352 (20.4)	22 (18.3)	0.632
Diabetes mellitus, *n* (%)	216 (12.5)	16 (13.3)	0.807
Accompanying symptoms, *n* (%)			
Otalgia	177 (10.3)	16 (13.3)	0.304
Dizziness	42 (2.4)	2 (1.6)	0.59
Tinnitus	158 (9.1)	8 (6.6)	0.351
Hyperacusis	106 (6.1)	6 (5.0)	0.605
Hearing disturbance	30 (1.7)	2 (1.6)	0.949

## Data Availability

Data was collected during the study in Kyung Hee Medical Center.
